# Clinical Utility and Cost‐Effectiveness of Pretreatment *NUDT15* Pharmacogenetic Testing to Prevent Thiopurine‐Induced Myelosuppression: A Genotype‐First Reverse Phenotyping Cohort Study Within the UK NIHR Inflammatory Bowel Disease Bioresource

**DOI:** 10.1111/apt.70232

**Published:** 2025-06-23

**Authors:** Christopher Roberts, Jaime Peters, Aleksejs Sazonvos, Neil Goodman, Mohmmed Sharip, Rebecca Smith, Maria Bishara, Claire Bewshea, Simeng Lin, Neil Chanchlani, Phoebe Hodges, Fakhirah Badrulhisham, Aamir Saifuddin, Sean Carlson, Andrea Centritto, Alexandra Marley, Muhammad Saad, Karishma Sethi‐Aora, Laura White, Alaa Abdelmeguid, Laetitia Pele, Shaji Sebastian, Christian Selinger, Peter Irving, Laura Fachal, Gareth Walker, Rachel Palmer, Nick Kennedy, Jayne Houghton, Mary Doona, Christopher Lamb, Chris Hyde, Miles Parkes, James Goodhand, Tariq Ahmad

**Affiliations:** ^1^ University of Exeter Inflammatory Bowel Disease and Pharmacogenetics Research Group Exeter UK; ^2^ Department of Gastroenterology Royal Devon University Healthcare NHS Foundation Trust Exeter Devon UK; ^3^ Exeter Test Group, Department of Health and Community Sciences, The Medical School University of Exeter Exeter UK; ^4^ Wellcome Sanger Institute Hinxton, Cambridgeshire UK; ^5^ Exeter Genomics Laboratory Royal Devon University Healthcare NHS Foundation Trust Exeter Devon UK; ^6^ Department of Gastroenterology Cambridge University Hospitals NHS Foundation Trust Cambridge UK; ^7^ Department of Medicine University of Cambridge Cambridge UK; ^8^ Department of Gastroenterology St Marks Hospital and Academic Institute, Gastroenterology London UK; ^9^ Department of Gastroenterology Bart's Health NHS Trust London UK; ^10^ Department of Gastroenterology Guy's and St Thomas' NHS Foundation Trust London UK; ^11^ Department of Gastroenterology Royal Wolverhampton NHS Trust Wolverhampton UK; ^12^ Department of Gastroenterology Northern Care Alliance NHS Foundation Trust (Bury Care Organisation) Salford UK; ^13^ Department of Gastroenterology Hull University Teaching Hospitals NHS Trust Hull UK; ^14^ National Institute for Health and Care Research IBD BioResource for Translational Research Cambridge UK; ^15^ Leeds Gastroenterology Institute, Leeds Teaching Hospitals NHS Trust Leeds UK; ^16^ School of Immunology and Microbial Sciences King's College London London UK; ^17^ Department of Gastroenterology and Hepatology Royal Brisbane and Women's Hospital Brisbane Brisbane Queensland Australia; ^18^ NHS Southwest Genomic Medicine Service Alliance, Bristol Genetics Laboratory Southmead Hospital Bristol UK; ^19^ Department of Gastroenterology Newcastle upon Tyne Hospitals NHS Foundation Trust Newcastle upon Tyne UK; ^20^ Translational & Clinic Research Institute, Faculty of Medical Sciences Newcastle University Newcastle upon Type UK

**Keywords:** cost‐effectiveness, expressivity, inflammatory bowel disease, myelosuppression, *NUDT15*, penetrance, pharmacogenetic testing, variant pathogenicity

## Abstract

**Background:**

The clinical utility and cost‐effectiveness of pre‐thiopurine *NUDT15* pharmacogenetic testing in European and admixed populations are unknown.

**Aims:**

To report the prevalence, penetrance, expressivity, and pathogenicity of *NUDT15* variant allele carriage and the diagnostic accuracy and cost‐effectiveness of an inexpensive loop‐mediated isothermal amplification (LAMP) test.

**Methods:**

Retrospective case‐matched cohort study of rates of severe myelosuppression in patients with and without loss‐of‐function *NUDT15* variant allele(s) treated with a thiopurine.

**Results:**

Overall, 1.3% of Europeans and 11.7% of South Asians carried a variant allele. Severe myelosuppression was associated with *NUDT15* variant allele carriage (11.3% vs 0.95%; *p* < 0.001). Carriage of a single *3, *6 or *9 variant allele was associated with a shorter time to severe myelosuppression. Numbers needed to genotype to prevent myelosuppression in Europeans and South Asians were 786 and 23. Variant calling using the LAMP assay was fully concordant with Sanger sequencing (*n* = 154). It improved the safety of thiopurine dosing and was cost‐effective when used to reduce the frequency and cost of thiopurine blood monitoring for patients without risk variants.

**Conclusion:**

We recommend *TPMT* and *NUDT15* genetic testing in patients of Asian and admixed ancestry. In Europeans, reflex *NUDT15* testing should be considered in patients with reduced TPMT activity or loss‐of‐function genotype. Thiopurines should be avoided in patients with > 1 *NUDT15* variant allele and in patients with both *NUDT15* and *TPMT* variant alleles. In patients with a single *NUDT15* variant allele, we recommend thiopurine dose reduction (< 1 mg/kg/day) and intensified blood test monitoring.

## Graphical abstract

Carriage of any loss of function *NUDT15* varaint is associated with an increased risk of thiopurine‐induced myelosuppression. Pre‐treatment genotype testing of *NUDT15*.

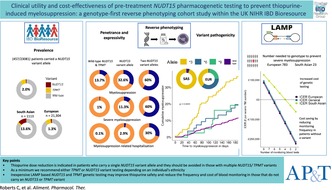

## 1 Introduction

Thiopurine‐induced myelosuppression is common and typically occurs within 6 months of commencing azathioprine, mercaptopurine or tioguanine [1]. Whilst most patients are asymptomatic, severe myelotoxicity, opportunistic infections and death have been reported [1].

In patients of European ancestry, loss of function variants in thiopurine methyltransferase (*TPMT*) account for 25% of cases of myelosuppression [2]. Reduced TPMT enzyme activity leads to an excess of unmethylated thioinosine monophosphate metabolites, which, when incorporated into DNA and RNA, triggers apoptosis. International guidelines recommend pre‐treatment testing of TPMT enzyme activity or *TPMT* genotype testing to guide thiopurine dosing and to mitigate the risk of myelosuppression [3, 4].

In patients of East Asian ancestry, myelosuppression is common, but *TPMT* variant carriage is rare. In 2014, an association between myelosuppression and variants in a second, previously unknown gene, *nudix hydrolase 15* (*NUDT15*) carried by 23% of this population was reported [5, 6]. Subsequent studies revealed that NUDT15 functions as a nucleoside diphosphatase, converting thioguanine triphosphate (TGTP) into the less toxic thioguanine monophosphate [4, 7]. Variants in *NUDT15* lead to defective activity and an increase of TGTP metabolites for DNA synthesis, leading to excess levels of DNA‐incorporated thioguanines [8]. In East Asian populations, inexpensive single variant genetic testing is cost‐effective at reducing the incidence of myelosuppression [9, 10, 11, 12]. Similarly, in South Asians, *NUDT15* testing has been adopted and reduces the incidence of myelosuppression [13].

Using a retrospective, phenotype‐first approach, we recruited patients of European ancestry with IBD and a history of severe myelosuppression and showed that carriage of a loss of function *NUDT15* variant was associated with a 27‐fold increased risk of myelosuppression [14]. Our previous study design did not permit an estimate of variant allele penetrance, and we therefore could not estimate the cost‐effectiveness of *NUDT15* pre‐treatment testing in this population to prevent myelosuppression.

In the United Kingdom (UK), because of the costs and predicted numbers of tests for patients with immune‐mediated inflammatory disease, pre‐treatment *NUDT15* testing is currently only available to patients with acute lymphoblastic leukaemia. Herein, to inform recommendations for *NUDT15* genotype‐based prescribing in all patients, we have undertaken a series of four related studies based on carriage of *NUDT15* variants in patients recruited to the UK National Institute for Health and Care Research (NIHR) IBD Bioresource.

We sought to define the:

1. Prevalence of *NUDT15* variants in the IBD Bioresource.
2. Penetrance, expressivity and variant pathogenicity of *NUDT15* variant carriage in patients treated with a thiopurine.
3. Diagnostic accuracy of a novel inexpensive loop‐mediated isothermal amplification testing (LAMP) *NUDT15* pharmacogenetic test.
4. Cost‐effectiveness of pre‐treatment testing for *NUDT15* with the LAMP‐based assay.

## 2 Methods

### 2.1 Study Design

We conducted a retrospective case‐matched cohort study to evaluate the rates of severe myelosuppression in patients with and without a loss of function *NUDT15* variant treated with a thiopurine.

### 2.2 Participants and Matching

Patients were identified from the UK NIHR IBD Bioresource, hereafter referred to simply as ‘IBD Bioresource’ https://www.ibdbioresource.nihr.ac.uk/ [15]. The IBD Bioresource is a panel of recallable patients with IBD who were recruited from more than 100 hospitals from across the UK between 2017 and 2024. It comprises genetic and phenotype data for more than 25,000 unselected consecutive patients' representative of the wider UK IBD population [15]. At enrolment to the IBD Bioresource, detailed phenotypic information was collected and a blood sample drawn for DNA extraction which was subsequently analysed by whole genome sequencing (WGS) or whole exome sequencing (WES) at the Wellcome Sanger Institute, Cambridge, UK. For full genetic methods see Appendix S2. Data were extracted from the IBD Bioresource on 19/02/2023.

We included all patients who carried a confirmed or suspected loss‐of‐function *NUDT15* variant (*2, *3, *4, *5, *6 and *9—Figure S1) irrespective of history of thiopurine treatment and *TPMT* status to define *NUDT15* variant carriage rate. The prevalence of *NUDT15* variant carriage is reported as the proportion of individuals who carry one or more variants and is stratified by genetic ancestry.

Patients without loss of function *NUDT15* or *TPMT* variant alleles with a history of past or current thiopurine treatment recorded in the IBD Bioresource were identified. After matching on genetic ancestry and recruiting site, three patients were randomly selected for each case with a loss of function *NUDT15* variant. Matching was undertaken by the central IBD Bioresource team who were blinded to myelosuppression outcomes and selected patients were identified to research sites without disclosing genotypes.

### 2.3 Outcomes

Between June and November 2023, 89 study sites were asked to confirm thiopurine exposure and complete a purpose‐designed data capture form for patients treated with a thiopurine.

Myelosuppression was defined as a white cell count (WCC) < 3.5 × 10^9^/L or a neutrophil count < 2.0 × 10^9^/L that occurred within 6 months of achieving the maximum thiopurine dose, or in the absence of blood test results, a decision to dose‐reduce or withdraw the thiopurine due to myelosuppression. Severe myelosuppression was defined as a WCC < 2.5 × 10^9^/L or neutrophil count < 1.0 × 10^9^/L and a decision to either reduce or withdraw the thiopurine.

Penetrance was defined as the proportion of individuals carrying a *NUDT15* variant treated with a thiopurine who had an episode of myelosuppression. Expressivity was defined as the proportion of individuals who had an episode of severe myelosuppression and was further stratified by the need for hospital admission due to myelosuppression. Sensitivity analyses of penetrance and expressivity in patients carrying a single *NUDT15* variant was stratified by *3, *6, *9 carriage to define variant pathogenicity.

### 2.4 Variables

Variables extracted from the IBD Bioresource included: demographics (age at thiopurine commencement, sex and genetic ancestry) and IBD history (disease type, location and behaviour and smoking status). Variables completed by local sites included: thiopurine treatment course (TPMT enzyme activity, drug, weight‐adjusted azathioprine equivalent dose [mercaptopurine weight‐adjusted doses were doubled to calculate an equivalent azathioprine weight‐adjusted dose], allopurinol usage, concomitant IBD medications and baseline white cell and neutrophil counts), myelosuppression history (lowest white cell and neutrophil counts in the 6 months after the maximum thiopurine dose was first achieved) and complications related to myelosuppression (hospitalisation, length of stay, ICU admission and use of G‐CSF [granulocyte‐colony stimulating factor]).

### 2.5 Diagnostic Accuracy of a Novel Inexpensive Loop‐Mediated Isothermal Amplification Testing (LAMP) NUDT15 Pharmacogenetic Test

To allow reporting of actionable genetic findings patients with a confirmed or suspected loss‐of‐function *NUDT15* variant were offered confirmatory Sanger sequencing using DNA extracted from a new blood sample. This was carried out in the UK accredited laboratory the accredited Exeter genomics laboratory https://www.exeterlaboratory.com/. Using surplus whole blood, we report the diagnostic accuracy statistics of the LAMP Human *NUDT15* deficiency KIT (LC‐NUDT15‐LP) https://lacar‐mdx.com/kit/pharmacogenetics/nudt15, using the results of our Sanger sequencing as our gold standard. Blood from patients of European ancestry attending routine outpatient appointments in Exeter was used as negative controls.

### 2.6 Genetic Methods

Whole genome and exome sequencing conducted at the Wellcome Sanger Institute, Cambridge, UK. Confirmatory Sanger sequencing and LAMP‐based *NUDT15* genotyping was undertaken at the Exeter genomics laboratory https://www.exeterlaboratory.com/.

Full methods of the Sanger sequencing and LAMP based genotyping are detailed in the supplement see appendix S2.

### 2.7 Cost‐Effectiveness Methods

We conducted a cost‐effectiveness analysis of pre‐treatment *NUDT15* testing using decision tree modelling. We compared our current practice, TPMT enzymatic activity testing (strategy 1), against three alternative strategies:

1. TPMT enzymatic activity and *NUDT15* LAMP based testing (strategy 2)
2. *TPMT* and *NUDT15* LAMP based testing (strategy 3)
3. No enzymatic or genetic testing (strategy 4) where thiopurines are avoided, and alternative treatments (such as vedolizumab, adalimumab, infliximab) are utilised.

The decision trees are illustrated for each of the four strategies in Figures S11–S13.

Each node of the decision tree represents an event (e.g., TPMT enzyme test), and probabilities are assigned to the potential outcomes (branches) of each node (e.g., prevalence of normal, intermediate or no TPMT activity). Analyses are conducted for the general UK IBD population, with sensitivity analyses for both European and South Asian subgroups. Probabilities at the nodes are detailed (Table 1) for all strategies and populations. The probability of stopping thiopurine treatment after a severe thiopurine induced myelosuppression (TIM) episode depends on whether the patient required hospitalisation and their *TPMT* and *NUDT15* status. Patients who did not require hospitalisation and had normal TPMT enzyme activity (in strategy 1), normal TPMT enzyme activity and *NUDT15* wild type (in strategy 2), *TPMT* wild type and *NUDT15* wild type (in strategy 3) have a 25% probability of stopping thiopurine treatment after resolution of their myelosuppression. In all other situations, 100% of patients would stop thiopurine treatment after a severe TIM episode.

**TABLE 1:** Proportion of patients in cost‐effectiveness analysis in each strategy and the probability of severe myelosuppression.

*TPMT* and *NUDT15* status	General population	European ancestry	South Asian ancestry
**Proportion of population with IBD by *TPMT* and *NUDT15* status**			
*TPMT* information only (Current practice, strategy 1)			
*TPMT* wild‐type/normal activity	0.909	0.906	0.974
*TPMT* heterozygous/intermediate activity	0.089	0.091	0.026
*TPMT* homozygous/no function	0.002	0.003	0.000
*TPMT* and *NUDT15* information (*NUDT15* testing, strategies 2 and 3)			
*TPMT* wild‐type + *NUDT15* wild‐type	0.890	0.894	0.841
*TPMT* wild‐type + *NUDT15* heterozygous	0.018	0.012	0.124
*TPMT* heterozygous + *NUDT15* wild‐type	0.088	0.090	0.023
*TPMT* homozygous or *NUDT15* homozygous or both	0.004	0.004	0.012
**Probability of severe myelosuppression**			
*TPMT* wild‐type/normal activity	1.12%	1.08%	1.98%
*TPMT* heterozygous/intermediate activity	2.07%	2.07%	3%
*TPMT* wild‐type/normal activity and *NUDT15* wild‐type	0.95%	1.01%	0.51%
*TPMT* wild‐type/normal activity + *NUDT15* heterozygous	9.52%	6.87%	11.94%
*TPMT* heterozygous/intermediate activity + *NUDT15* wild‐type	1.70%	1.70%	1.70%
Probability of hospitalisation given severe TIM	23.5%		

The primary health outcome is avoidance of severe TIM, with quality‐adjusted life‐years (QALYs) as a secondary health outcome. We assume a utility of 0.70 for patients who are not experiencing a severe TIM [16], a disutility of 0.292 for a severe TIM [17] (for a length of 38.5 days [14]), and a utility of 0 for severe TIM [18] requiring hospitalisation (assumed for a length of 7 days). As no deaths from severe TIM were observed in our cohort, the relatively young population, and the short model time‐horizon; we assumed no deaths in this model. In our cost‐effectiveness models we included data from our primary analysis of the IBD Bioresource data, our previous work, peer‐reviewed publications and expert opinion.

We included costs (UK pound sterling £, 2024) of *NUDT15* and *TPMT* testing, recommended frequency of blood test monitoring [3] for all strategies, costs associated with the treatment of myelosuppression and of alternative treatments. We assumed that 40% of patients that had thiopurines had them in combination with infliximab. These costs were taken from the British National Formulary and the national schedule of National Health Service (NHS) costs. The costs associated with an episode of severe myelosuppression includes the costs associated with hospital admission, time and costs of intensified blood test monitoring and the costs of alternative treatment. We assumed that no patients stopped the thiopurine due to lack of response, over the 1‐year time‐horizon. We also report the cost‐effectiveness of reduced frequency of blood test monitoring in patients without carriage of a *TPMT* and *NUDT15* variant.

### 2.8 Statistical Analysis

Because the penetrance of *NUDT15* in Europeans is poorly defined, we sought to include all patients with a loss‐of‐function *NUDT15* variant in the IBD Bioresource.

Data were entered electronically into a purpose‐designed REDCap case report form hosted at the IBD Bioresource. Statistical analyses were undertaken in R 4.4.2 (R Foundation for Statistical Computing, Vienna, Austria). All tests were two‐tailed and values of *p* < 0.05 were considered significant. Participants with incomplete clinical data were included in analyses where data were available, with the denominator for each variable clearly specified. Continuous data are reported as median and interquartile range (IQR) and discrete data are presented as numbers and percentages and 95% confidence intervals (95% CI) and were compared using Mann–Whitney *U* tests and Fisher's exact tests, respectively.

Cox‐proportional hazard ratios were used to compare time to severe myelosuppression in patients with *NUDT15* variants and those with wild‐type *NUDT15* and *TPMT*. Sensitivity analyses were undertaken in *NUDT15* heterozygotes to identify the pathogenicity of individual variants and determine the impact of azathioprine equivalent weight‐adjusted doses.

The number needed to genotype to prevent one case of severe myelosuppression is the number needed to harm divided by the prevalence of any loss‐of‐function *NUDT15* variant in the population. The number needed to harm represents the number of patients with a *NUDT15* variant who, when treated with a thiopurine, would lead to one instance of severe myelosuppression.

Cost‐effectiveness analyses were conducted from the perspective of the NHS. The primary cost‐effectiveness outcome was the incremental cost‐effectiveness ratio (ICER). The secondary cost effectiveness outcome was QALYs.

### 2.9 Ethics

The IBD Bioresource operates under ethical approval granted by the Health Research Authority and the NHS Research Ethics Committee (reference number 15/EE/0286 and 22/EE/0230). Additionally, this project has been reviewed and approved by the Data Access Committee at the IBD Bioresource, receiving the reference number DAA151.

## 3 Results

**FIGURE 1:**
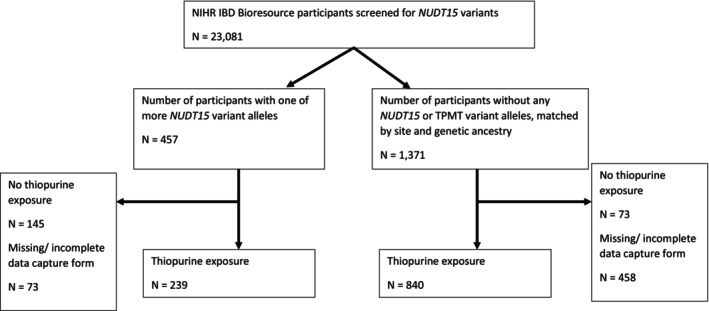
Study profile: A flowchart demonstrating patient disposition throughout the study.

Patient disposition through the study is shown in Figure 1.

### 3.1 Prevalence of *NUDT15* Variants in the IBD BioResource

*NUDT15* variant alleles were observed in 2.0% (457/23081; 95% CI 1.8%–2.2%) of participants in the NIHR IBD Bioresource with high quality genetic sequence calls at both *NUDT15* and *TPMT*. 27/23081 (0.1% 95% CI 0.07–0.17) participants had both *NUDT15* and *TPMT* variant alleles.

Variant alleles in *NUDT15* were more common in patients of East Asian (21.7% [10/46] 95% CI 10.9%–36.3%, *p* < 0.0001) and South Asian ancestry (13.6% [151/1113] 95% CI 11.6–15.7, *p* < 0.001), compared to those of European ancestry (1.3% [271/21304] 95% CI 1.1%–1.3%).

Amongst carriers of a *NUDT15* variant allele: 90.2% (412/457; 95% CI 87.0–97.2) were heterozygous for a *NUDT15* variant allele and carried wild‐type *TPMT*; 3.9% (18/457; 95% CI 2.3–6.1) carried two variant *NUDT15* alleles and wild‐type *TPMT*; and 5.9% (27/457; 95% CI 3.9–8.5) had a single *NUDT15* variant allele and one or more *TPMT* variant alleles.

In participants of South Asian ancestry, 94.7% (143/151; 95% CI 89.8–97.6) carried the *3 missense *NUDT15* variant. In participants of European ancestry, 38.0% (103/271; 95% CI 32.2–44.1) carried the *3 missense variant; 35.8% (97/271; 95% CI 30.0–41.8) carried the *6 insertion variant and 25.1% (68/271; 95% CI 20.0–30.7) carried the *9 deletion variant (Figure S2). Too few participants from other genetic ancestries were identified to allow meaningful estimates of variant carriage to be made.

### 3.2 Penetrance, Expressivity and Variant Pathogenicity of *NUDT15* Variant Carriage

Completed case report forms were returned for 84.0% (384/457) of study patients with *NUDT15* variants, of which treatment with a thiopurine was confirmed in 62.2% (239/384). Amongst matched patients without *NUDT15* and *TPMT* variant allele carriage, case report forms were returned for 66.6% (913/1371), of which 92.0% (840/913) had been treated with a thiopurine.

Baseline characteristics of study patients treated with a thiopurine are summarised in Table 2. There were no significant differences in age, sex, disease type, and smoking status between patients with and without *NUDT15* variant alleles. Similar proportions of patients in both groups were treated with azathioprine and mercaptopurine, and there were no differences in the median azathioprine equivalent weight‐adjusted doses between groups. Concomitant use of infliximab, but no other IBD medications, was more common in those without a loss of function *NUDT15* variant allele. Baseline white cell and neutrophil counts were lower in patients who carried a *NUDT15* variant allele.

**TABLE 2:** Baseline characteristics of study participants.

Data source	Categotry		Wild type *NUDT15*	Loss of function *NUDT15* variant alleles	*p*
NIHR IBD BioResource data	Age at thiopurine commencement (years)		33 (24–46)	33 (25–49)	N/S
	Sex	Male	52.6% (442/840)	51.5% (123/239)	N/S
		Female	47.4% (398/840)	48.5% (116/239)	
	Genetic ancestry	African	4.6% (39/840)	1.7% (4/239)	< 0.001
		East Asian	0.6% (5/840)	2.1% (5/239)	
		European	70.8% (595/840)	60.7% (145/239)	
		South Asian	23.3% (196/840)	31.8% (76/239)	
		Other	0.6% (5/840)	3.8% (9/239)	
	Smoking history	Never smoked	54.9% (461/840)	56.9% (136/239)	N/S
		Smoked at diagnosis	18.7% (157/840)	18.8% (45/239)	
		Quit before diagnosis	19.3% (162/840)	20.5% (49/239)	
		Unknown	6.2% (52/840)	2.5% (6/239)	
	Diagnosis	Crohn's disease	46.1% (387/840)	51.5% (123/239)	N/S
		Ulcerative colitis	50.6% (425/840)	45.6% (109/239)	
		IBD‐U	3.3% (28/840)	2.9% (7/239)	
From sites	Thiopurine	Azathioprine	92.6% (775/837)	92.0% (219/238)	N/S
		Mercaptopurine	7.4% (62/837)	8.0% (19/238)	
	Median azathioprine equivalent weight adjusted dose of thiopurine (mg/kg/day)		1.87 (1.42–2.11)	1.79 (1.24–2.06)	N/S
	Low dose thiopurine (< 1 mg/kg/day)		13.1% (74/565)	18.4% (23/125)	N/S
	Concurrent IBD drugs at the time of thiopurine commencement	Steroids	45.8% (385/840)	42.3% (101/239)	N/S
		Budesonide	4.2% (35/840)	2.5% (6/239)	N/S
		5‐ASA	38.2% (321/840)	38.9% (93/239)	N/S
		Methotrexate	0.2% (2/840)	0.8% (2/239)	N/S
		Ciclosporin	1.8% (15/840)	2.1% (5/239)	N/S
		Infliximab	13.9% (117/840)	8.0% (19/239)	0.015
		Adalimumab	1.9% (16/840)	2.5% (6/239)	N/S
		Golimumab	0.2% (2/840)	0%	N/S
		Vedolizumab	0.2% (2/840)	0.8% (2/239)	N/S
		Ustekinumab	0.2% (2/840)	0%	N/S
	Allopurinol usage		5.8% (42/720)	3.0% (6/199)	N/S
	Baseline WCC count (×10^9^/L)		9.0 (7.0–11.3)	8.1 (6.4–11.0)	0.02
	Baseline neutrophil count (×10^9^/L)		6.0 (4.4–8.3)	5.3 (3.9–7.6)	0.01

*Note:* Data is presented as *n*/*N* (%), or median (IQR) unless otherwise stated. Baseline characteristics broken down by *NUDT15* variant status.

Both myelosuppression and severe myelosuppression occurring within 6 months of the maximal thiopurine dose was more common in patients carrying a *NUDT15* variant allele than patients without *NUDT15* or *TPMT* variants (32.6% [78/239]; 95% CI 27.0–38.8 vs. 13.7% [115/840]; 95% CI 11.5–16.2, OR 3.1 [2.2–4.3, *p* < 0.001]) and (11.3% [27/239]; 95% CI 7.9–15.9 vs. 0.95% [8/840]; 95% CI 0.48–1.87, OR 13.3 [6.2–31.6, *p* < 0.001]), respectively as shown in Table 3. A further episode of myelosuppression was more common in patients rechallenged with a thiopurine in those with a loss of function *NUDT15* variant than those without a loss of function *NUDT15* variant (47.1% [8/17] vs. 12.5% [2/16], OR 6.2 [1.2–47.9, *p* = 0.04]). After 6 months of reaching the maximum thiopurine dose, more patients who carried a *NUDT15* variant had stopped the thiopurine than patients who did not carry a *NUDT15* or *TPMT* variant allele (49.4% [118/239] 95% CI 43.1–55.7 vs. 36.3% [305/840] 95% CI 33.1%–39.6%, *p* < 0.001).

**TABLE 3:** Table comparing the rates of myelosuppression, severe myelosuppression, and myelosuppression related hospitalisation, stratified by number of variants and by the specific allele carried.^a^

	NUDT15 genotype	Penetrance	Expressivity	
		Myelosuppression (WCC < 3.5 × 10^9^/L OR neutrophil count < 2.0 × 10^9^/L)	Severe myelosuppression (WCC < 2.5 × 10^9^/L OR neutrophil count < 1.0 × 10^9^/L)	Myelosuppression related hospitalisation
	Wild‐type	13.7% (115/840)	1% (8/840)	0.1% (1/840)
Penetrance	Carriage of any *NUDT15* variant	32.6% (78/239) OR 3.1 (2.2–4.3, *p* < 0.001)	11.3% (27/239) OR 13.3 (6.2–31.6, *p* < 0.001)	2.9% (7/239) OR 7.0 (1.0–142.6, *p* = 0.09)
	*NUDT15* single variant	31.9% (67/210) OR 3.0 (2.1–4.2, *p* < 0.001)	9.5% (20/210) OR 11.0 (4.9–26.8, *p* < 0.001)	1.9% (4/210) OR 4.4 (0.5–94.1, *p* = 0.2)
	*NUDT15* multiple variants	60% (6/10) OR 9.5 (2.7–37.5, *p* < 0.001)	60% (6/10) OR 156.0 (37.9–724.1, *p* < 0.001)	30% (3/10) OR 33.0 (2.2–1405.9, *p* = 0.02)
Variant pathogenicity	*3 heterozygotes	39.8% (47/118) OR 4.2 (2.7–6.3, *p* < 0.001)	11.0% (13/118) OR 12.9 (5.3–33.2, *p* < 0.001)	2.5% (3/118) OR 4.1 (0.4–92.4, *p* = 0.3)
	*6 heterozygotes	20.8% (11/53) OR 1.7 (0.8–3.2, *p* = 0.2)	9.4% (5/53) OR 10.8 (3.2–33.8, *p* < 0.001)	0% 0/53 —
	*9 heterozygotes	22.2% (8/36) OR 1.8 (0.8–3.9, *p* = 0.2)	5.6% (2/36) OR 6.1 (0.9–25.6, *p* = 0.03)	2.8% (1/36) —

*Note:* Data is presented as *n*/*N* (%) and by the odds ratio (OR) of the event occurring compared to a patient with no loss of function *NUDT15* variants termed wild‐type *NUDT15*.

^a^ Variant pathogenicity assessment was only carried out in patients that were *NUDT15* heterozygotes.

Sensitivity analyses in patients with a single *NUDT15* variant showed an azathioprine equivalent weight‐adjusted dose response with risk of severe myelosuppression (Table 4). Even amongst patients treated with a thiopurine adjusted dose of less than 1 mg/kg/day, rates of severe myelosuppression were higher in patients with a single *NUDT15* variant than in patients without *NUDT15* and *TPMT* variant alleles (6.3% [1/16] 95% CI 0.3–32.3 vs. 0% [0/77] 95% CI 0–5.9, OR 10.9 [4.9–26.8, *p* < 0.001]). In those patients with a single *NUDT15* variant that were rechallenged with a dose of less than 1 mg/kg/day, there were no cases of further myelosuppression (0/4).

**TABLE 4:** Rates of any and severe myelosuppression in those with a single *NUDT15* variant allele and no loss of function *NUDT15* variant stratified by weight‐adjusted azathioprine‐equivalent dose per day.

	All myelosuppression (WCC < 3.5 × 10^9^/L OR neutrophil count < 2.0 × 10^9^/L)		Severe myelosuppression (WCC < 2.5 × 10^9^/L OR neutrophil count < 1.0 × 10^9^/L)	
	Single *NUDT15* variant allele	No loss of function *NUDT15* allele	Single *NUDT15* variant allele	No loss of function *NUDT15* allele
< 1.0 mg/kg	12.5% (2/16)	9.1% (7/77)	6.3% (1/16)	0% (0/77)
1.0–2.0 mg/kg	38.1% (24/63)	15.7% (45/286)	11.1% (7/63)	0.7% (2/286)
> 2.0 mg/kg	38.9% (14/36)	15.8% (32/202)	16.7% (6/36)	2.0% (4/202)
*p*	0.13	0.31	0.61	0.36

*Note:* Data is presented as *n*/*N* (%).

No differences were observed between groups in rates of hospitalisation due to myelosuppression. 75% (6/8) required treatment with G‐CSF. These patients had a median [IQR] length of stay of 5 [8.5] days and none were admitted to the intensive care unit or died.

Patients who were *NUDT15* heterozygotes with alleles *3, *6 or *9 had increased rates of any and severe myelosuppression when compared to patients without *NUDT15* or *TPMT* variants, as shown in Table 5. Those with *3 had higher rates of myelosuppression, but there was no difference in the rates of severe myelosuppression. The highest rates of any and severe myelosuppression were observed in patients with more than one *NUDT15* variant allele: 60% (6/10) and 40% (4/10). The rates of any and severe myelosuppression in patients with a single *NUDT15* and *TPMT* variant allele were 26.3% (5/19) and 5.3% (1/19). Patients with both *TPMT* and *NUDT15* variant alleles were treated with lower weight‐adjusted doses of a thiopurine compared to patients with wild‐type *NUDT15* and *TPMT* (0.65 mg/kg/day vs. 1.88 mg/kg/day [*p* < 0.0001]).

**TABLE 5:** – Rates of any and severe myelosuppression in those with a single *NUDT15* variant allele stratified by *NUDT15* variant alleles.

*NUDT15* heterozygote variant	Rate of myelosuppression (WCC < 3.5 × 10^9^/L OR neutrophil count < 2.0 × 10^9^/L)	Rate of severe myelosuppression (WCC < 2.5 × 10^9^/L OR neutrophil count < 1.0 × 10^9^/L)
*2	NA	NA
*3	39.8% (47/118)	11.0% (13/118)
*4	0% (0/1)	0% (0/1)
*5	50% (0/1)	0% (0/2)
*6	20.8% (11/53)	9.4% (5/53)
*9	22.2% (8/36)	5.6% (2/36)
*p*	0.03	0.80

*Note:* Data is presented as *n*/*N* (%).

Univariable factors associated with reduced time to severe myelosuppression are shown in Table 6.

**TABLE 6:** Univariable predictors for a shorter time to severe myelosuppression compared to patients without any loss of function *NUDT15* variants using cox proportional hazard ratios.

Model		Hazard ratio	95% Confidence limits	*p*
Any *NUDT15* variant		12.4	5.6–27.4	< 0.0001
Variant status	*NUDT15* single variant allele	10.7	4.7–24.3	< 0.0001
	*NUDT15* 2 or more variant allele	89.2	29.5–269.3	< 0.0001
	Single *NUDT15* and *TPMT* variant allele	5.7	0.7–45.6	N/S
Diagnosis	Ulcerative colitis	1.5	0.7–2.9	N/S
Thiopurine weight‐adjusted dose		1.5	0.7–3.3	N/S
*NUDT15* Heterozygotes	*3	13.2	5.5–31.8	< 0.0001
	*6	10.2	3.3–31.1	< 0.0001
	*9	5.9	1.2–27.6	0.03
Prior IBD medications	Steroids	1.0	0.5–1.8	N/S
	5‐ASA	1.6	0.8–3.0	N/S
	Infliximab	0.7	0.2–2.3	N/S
	Adalimumab	1.5	0.2–10.8	N/S

**FIGURE 2:**
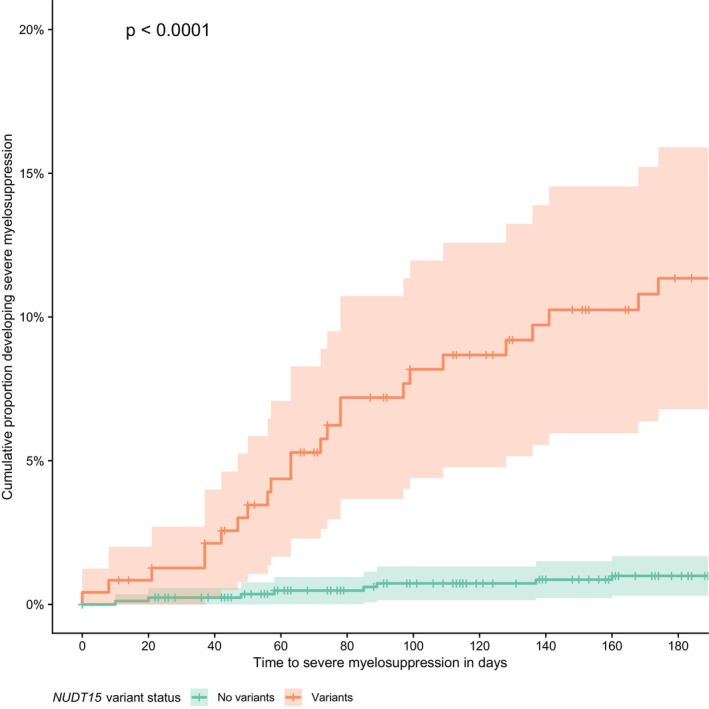
Time to severe myelosuppression based on *NUDT15* variant status. Severe myelosuppression defined as an episode where WCC < 2.5 × 10^9^/L or neutrophil count < 1.0 × 10^9^/L and a decision to either dose reduce or stop the thiopurine.

Time to severe myelosuppression was shorter in patients with a *NUDT15* variant allele than in patients who did not carry a *NUDT15* and *TPMT* variant allele (HR 12.4 [95% CIs 5.6–27.4], *p* < 0.0001) (Figure 2). Similarly, the time to myelosuppression was shorter in patients with a *NUDT15* variant allele than in those with wild‐type *NUDT15* (HR 2.6 [95% CIs 1.9–3.4], *p* < 0.0001) (Figure S4). The shortest time to severe myelosuppression was observed in patients with 2 or more *NUDT15* variant alleles (HR 89.2 [95% CIs 29.5–269.3]) (Figure S5). No differences were observed in the time to myelosuppression and severe myelosuppression when variant carriage was stratified by specific *NUDT15* allele (Figures S6 and S7).

Overall, the number needed to genotype to prevent a single case of severe myelosuppression was 786 (95% CI 451‐3045) in patients of European ancestry and 23 (95% CI 16–36) in patients of South Asian ancestry.

### 3.3 Diagnostic Accuracy of the LAMP Human *NUDT15* Deficiency Test Kit

We tested the diagnostic accuracy of the Human *NUDT15* deficiency KIT (LC‐NUDT15‐LP) in 125 patients who opted for confirmatory Sanger sequencing and 29 negative controls. Six samples tested on the semi‐automated platform failed the quality control checks and were repeated manually. We observed complete concordance between Sanger sequencing and the LAMP based assay for *3, *6 and *9 variant allele carriage.

### 3.4 Cost‐Effectiveness of LAMP Based Genetic Testing of *NUDT15* Variant Allele Carriage

The estimated total costs and component costs for each strategy are illustrated (Table 7) alongside the percentage of severe TIM events (hospitalised and non‐hospitalised) for each strategy.

**TABLE 7:** Components of costs from severe myelosuppression based on different strategies.^a^

	Cost components			Total costs	% Cohort			Total % severe TIM events
	Testing	UC/CD treatment and monitoring	Treatment for severe TIM events		Severe TIM non‐hospitalised	Severe TIM hospitalised	Taking alternative treatment	
*Overall population*								
1. TPMT enzyme testing (Current practice)	£52	£1939	£15	£2007	0.92%	0.28%	0.86%	1.20%
2. TPMT enzyme and *NUDT15* genotyping	£86	£1908	£15	£2009	0.89%	0.27%	1.12%	1.17%
3. *NUDT15* and *TPMT* genotyping	£80	£1908	£15	£2004	0.89%	0.27%	1.12%	1.17%
4. Alternative treatment	£0	£4743	£0	£4743	0.00%	0.00%	100.00%	0.00%
*European ancestry sub‐population*								
1. TPMT enzyme testing (Current practice)	£52	£1939	£15	£2006	0.90%	0.27%	0.86%	1.17%
2. TPMT enzyme and *NUDT15* genotyping	£86	£1905	£15	£2005	0.87%	0.27%	0.97%	1.14%
3. *NUDT15* and *TPMT* genotyping	£80	£1905	£15	£2000	0.87%	0.27%	0.97%	1.14%
4. Alternative treatment	£0	£4811	£0	£4811	0.00%	0.00%	100.00%	0.00%
*South Asian ancestry sub‐population*								
1. TPMT enzyme testing (Current practice)	£52	£1934	£26	£2012	1.53%	0.47%	0.90%	2.01%
2. TPMT enzyme and *NUDT15* genotyping	£86	£1943	£25	£2054	1.49%	0.46%	2.87%	1.95%
3. *NUDT15* and *TPMT* genotyping	£80	£1943	£25	£2049	1.49%	0.46%	2.87%	1.95%
4. Alternative treatment	£0	£4646	£0	£4646	0.00%	0.00%	100.00%	0.00%

^a^ Ulcerative colitis (UC), crohn's disease (CD).

In the population overall, irrespective of ancestry, our current practice (strategy 1) of pre‐treatment TPMT enzymatic activity testing alone was associated with the highest number of severe myelosuppression events (Table 8). In contrast, not testing and avoiding thiopurines resulted in no severe thiopurine induced myelosuppression events, but was the most expensive strategy because of the use of more costly alternative treatments (strategy 4).

**TABLE 8:** Cost of *NUDT15* testing strategies where blood test monitoring remains unchanged at 8 tests in the first year of treatment.

Strategy	% severe TIM	QALYs	Total costs	Compared to current practice				
				Incremental severe TIM events	Incremental QALYs	Incremental costs	ICER (per severe TIM avoided)	ICER (per QALY gained)
*Overall population*								
1. TPMT enzyme testing (Current practice)	1.20%	0.69957	£2007	N/A				
2. TPMT enzyme and *NUDT15* genotyping	1.17%	0.69959	£2009	−0.04%	0.00001	£39	£108,888	£3.08 million
3. *NUDT15* and *TPMT* genotyping	1.17%	0.69959	£2004	−0.04%	0.00001	£34	£94,647	£2.67 million
4. Alternative treatment	0%	0.70000	£4743	−1.20%	0.00043	£2737	£227,610	£6.43 million
*European ancestry sub‐population*								
1. TPMT enzyme testing (Current practice)	1.17%	0.69959	£2006	N/A				
2. TPMT enzyme and *NUDT15* genotyping	1.14%	0.69960	£2005	−0.04%	0.00001	£36	£100,241	£2.83 million
3. *NUDT15* and *TPMT* genotyping	1.14%	0.69960	£2000	−0.04%	0.00001	£30	£85,917	£2.43 million
4. Alternative treatment	0%	0.70000	£4811	−1.17%	0.00041	£2805	£239,482	£6.77 million
*South Asian ancestry sub‐population*								
1. TPMT enzyme testing (Current practice)	2.01%	0.69929	£2012	N/A				
2. TPMT enzyme and *NUDT15* genotyping	1.95%	0.69931	£2054	−0.06%	0.00002	£76	£135,495	£3.83 million
3. *NUDT15* and *TPMT* genotyping	1.95%	0.69931	£2049	−0.06%	0.00002	£71	£126,455	£3.57 million
4. Alternative treatment	0%	0.70000	£4646	−2.01%	0.00071	£2634	£131,326	£3.71 million

Strategy 2 (TPMT enzyme activity and *NUDT15* genetic testing) and strategy 3 (*TPMT* and *NUDT15* genetic testing) were both associated with a reduction in severe myelosuppression events but also a significant increase in ICER per event avoided (£108,888 and £94,647, respectively) and QALY (£3.08 million and £2.67 million, respectively).

In the South Asian population, for all strategies, the ICER per event avoided and QALY was higher than in Europeans, reflecting the higher *NUDT15* carriage rate and increased likelihood of requiring more costly alternative drugs (Table 8).

**FIGURE 3:**
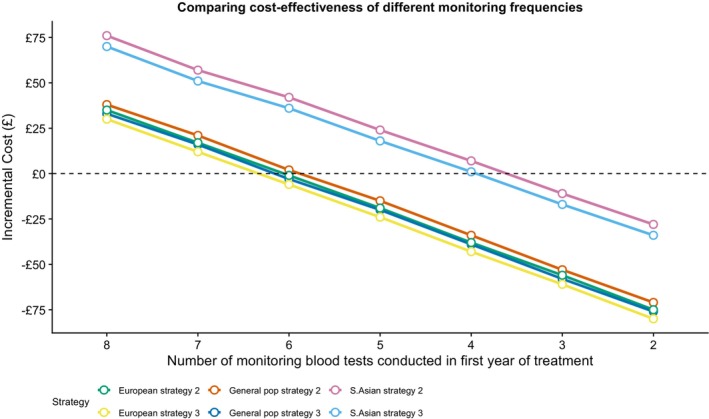
Incremental cost of *NUDT15* testing in addition to standard of care TPMT enzyme activity testing (strategy 2) or genetic testing (strategy 3) stratified by number of monitoring blood tests conducted in the first year of thiopurine treatment.

About 20% of the costs associated with thiopurine usage are related to the need for blood test monitoring (Table S2). In a sensitivity analysis, we modelled a reduction in the frequency of blood test monitoring in patients who do not carry *TPMT* and *NUDT15* variants. We demonstrated that *TPMT* and *NUDT15* genetic testing becomes more cost‐effective in the overall population when the number of costly tests in the first year is minimally reduced (Figure 3).

## 4 Discussion

### 4.1 Key Results and Interpretation

Thiopurines remain a key component of the therapeutic armamentarium for immune‐mediated inflammatory diseases and acute lymphoblastic leukaemia. In patients with IBD, they are a cheap and effective treatment for ulcerative colitis (UC) [3, 19] and are used to prevent immunogenicity to anti‐TNF therapies in both UC and Crohn's disease [20, 21].

Here, we have selected patients based on genotype rather than phenotype to overcome the ascertainment bias inherent to traditional phenotype‐first genetic studies. This has allowed us to more accurately and economically report the penetrance, expressivity and pathogenicity of rare variants using specific and consistent phenotypic assessments. Overall, about one‐third of patients with a *NUDT15* variant treated with a thiopurine developed myelosuppression.

Contrary to our previous report [14] but like other studies [22], severe myelosuppression was observed in about 11% of patients and opportunistic infections and hospitalisations were uncommon. We likely overestimated the risk of severe myelosuppression attributable to *NUDT15* variant carriage in our previous study because of recall/severity bias inherent to our retrospective case–control study design [14]. It is notable though that the median azathioprine‐equivalent dose in the affected cases was higher in our previous study than observed here. We confirmed here the previously reported additive risk of carriage of more than one *NUDT15* variant. We did not observe any significant differences in the penetrance and time‐to‐myelosuppression between *NUDT15* *3, *6, and *9. However, we acknowledge that this sub‐analysis may be underpowered due to the low frequency of *6 and *9 *NUDT15* alleles herein.

In patients who carried a single loss of function *NUDT15* variant, use of azathioprine equivalent doses of less than 1 mg/kg/day was safer than standard dosing but did not negate the risk of severe myelosuppression. Too few patients who carried *NUDT15* variant alleles and experienced myelosuppression following treatment with standard thiopurine doses were rechallenged with a lower dose to define whether dose reduction is a safe strategy.

To date, international recommendations do not mandate *NUDT15* testing in at‐risk populations, in part due to limited access to inexpensive testing [3, 4]. In contrast, TPMT genetic or enzyme activity testing has been widely implemented even in populations where *TPMT* variant alleles are less common. Arguably, in South and East Asians, based on *NUDT15* variant carriage rates, which exceed *TPMT* variant carriage rates in European populations, *NUDT15* genotype testing should be conducted in addition to TPMT testing. Patients of European and Asian mixed ethnicity require combined *TPMT* and *NUDT15* testing until further data are available. Overall, our analyses suggest that combined genetic testing in European and Asian patients is the most cost‐effective strategy. However, in patients of European ancestry, where access to *NUDT15* testing may be limited, reflex testing of *NUDT15* might be considered in patients with a single *TPMT* variant due to the potential catastrophic effects of thiopurine use in patients carrying both *TPMT* and *NUDT15* variant alleles [14, 23].

The LaCAR LAMP assay, which can be used as part of a panel of pharmacogenomic markers [24], is significantly less expensive than current genetic testing and here we have shown concordant variant calling with gold standard Sanger sequencing for *NUDT15* *3, *6, *9 testing. Even using this relatively inexpensive assay, because the penetrance of *NUDT15* variant carriage is low and hospitalisation is rare, pre‐treatment *NUDT15* testing was not cost‐effective. However, an often‐overlooked application of pharmacogenetic testing is its potential to reduce the need for monitoring tests in patients who do not carry risk variants. Here, in patients without a *NUDT15* variant, severe thiopurine‐induced myelosuppression and hospitalisation and opportunistic infection were uncommon. By modelling a reduction in the frequency of blood test monitoring in patients who do not carry *TPMT* and *NUDT15* variants, we demonstrated that *TPMT* and *NUDT15* genetic testing becomes cost‐effective in the overall population when the number of tests in the first year is modestly reduced. To mitigate the effects of rare *TPMT* and *NUDT15* variants that are not captured by the current LAMP test, we would recommend blood test monitoring at baseline, weeks 2, 6, 24, 36 and 52. Concerns about the costs of genetic testing will become even less relevant as whole exome and genome sequencing becomes widely available. The next challenge will be integrating pre‐existing genetic data into electronic health records and upskilling physicians to interpret results rather than whether or not to test [24].

We acknowledge the following limitations. Firstly, due to the retrospective nature of data collection, our findings are limited by interpretation bias and missingness, particularly when the thiopurine treatment was more historic. Secondly, by undertaking our study within the IBD Bioresource, we may have underestimated the rates of death associated with myelosuppression. Thirdly, in this study, patients were treated with a median azathioprine equivalent weight‐adjusted dose of 1.8 mg/kg/day, lower than the dose recommended in international IBD guidelines. Therefore, it is likely we will have underestimated the risk of myelosuppression in patients with both *TPMT* and *NUDT15* variants. Fourthly, because we undertook the study during the COVID‐19 pandemic, sites were asked to preferentially focus on data collection in patients who carried *NUDT15* variants and consequently the matching for ancestry was imperfect. Fifthly, unlike our previous pharmacogenomic studies, we did not independently adjudicate causality of myelosuppression, which may have inflated our estimates of the rates of myelosuppression. Sixthly, without parental genomes or long‐read sequences, it was not possible to determine whether two non‐identical heterozygote variants occurred on the same or opposite chromosomes. This phasing difficulty limited our ability to identify a small number of *NUDT15* compound heterozygous patients. For example, for patients who carried both a *3 and *6 variant, we were unable to confidently assign a heterozygote *2/*1 or compound heterozygote *3/*6 *NUDT15* genotype. Finally, too few patients of African and Hispanic ancestry were recruited to draw meaningful conclusions in these sub‐populations.

We acknowledge several limitations in our cost‐effectiveness analysis. Firstly, traditional cost‐effectiveness analyses are inherently susceptible to uncertainty around the assumptions used, such as the 1‐year time horizon. Secondly, due to a lack of data on the risk of myelosuppression in individuals with *TPMT* variants, we have relied on expert opinion. Thirdly, thresholds for determining the cost‐effectiveness of genetic testing are poorly defined. For example, the National Institute for Health and Care Excellence sets a threshold of £20,000–£30,000 per QALY; however, this varies depending on what a healthcare system can afford. Finally, the cost‐effectiveness of genetic testing is highly dependent on test costs, which continue to fall.

Our findings are likely to be generalisable to mixed ancestry populations across other disease indications in which thiopurines are used, including transplant medicine, neurology, renal medicine, and autoimmune hepatitis. Differences between our sensitivity analyses in South Asians and other cost‐effectiveness analyses from Japan and China are likely influenced by our inclusion of the more expensive alternative advanced therapies [9, 10, 12] and by more accurate analysis of the penetrance of *NUDT15* variant carriage, which is lower than previously reported.

## 5 Conclusions

We recommend *TPMT* and *NUDT15* genetic testing in patients of Asian and admixed ancestry. In Europeans, reflex *NUDT15* testing should be considered in patients with reduced TPMT activity or loss‐of‐function genotype.

Thiopurines should be avoided in patients who carry more than one *NUDT15* variant allele and in patients who carry both *NUDT15* and *TPMT* variant alleles. In patients who carry a single *3, *6 or *9 *NUDT15* variant allele, we recommend thiopurine dose reduction (< 1 mg/kg/day) followed by intensified blood test monitoring.

Inexpensive LAMP‐based *NUDT15* variant testing can be used to improve the safety of thiopurine dosing in patients with *TPMT* or *NUDT15* variant alleles and to reduce the frequency and substantial cost of thiopurine blood monitoring for most patients who do not carry risk variants.

## Author Contributions

**Christopher Roberts:** conceptualization, investigation, writing – original draft, methodology, validation, visualization, writing – review and editing, software, formal analysis, data curation, resources. **Jaime Peters:** investigation, writing – original draft, methodology, visualization, validation, writing – review and editing, software, formal analysis, data curation, conceptualization. **Aleksejs Sazonvos:** investigation, methodology. **Neil Goodman:** investigation, methodology, validation, formal analysis, software, data curation. **Mohmmed Sharip:** investigation, writing – review and editing, data curation. **Rebecca Smith:** data curation, conceptualization, writing – review and editing. **Maria Bishara:** investigation, software. **Claire Bewshea:** project administration, conceptualization, investigation, writing – review and editing, funding acquisition. **Simeng Lin:** conceptualization, writing – review and editing. **Neil Chanchlani:** conceptualization, writing – review and editing. **Phoebe Hodges:** investigation, writing – review and editing. **Fakhirah Badrulhisham:** investigation, writing – review and editing. **Aamir Saifuddin:** data curation, writing – review and editing. **Sean Carlson:** writing – review and editing, data curation. **Andrea Centritto:** writing – review and editing, data curation. **Alexandra Marley:** data curation, writing – review and editing. **Muhammad Saad:** writing – review and editing, data curation. **Karishma Sethi‐Aora:** writing – review and editing, data curation. **Laura White:** writing – review and editing, data curation. **Alaa Abdelmeguid:** data curation, writing – review and editing. **Laetitia Pele:** project administration, supervision. **Shaji Sebastian:** investigation, writing – review and editing, supervision. **Christian Selinger:** supervision, data curation, writing – review and editing. **Mary Doona:** data curation. **Christopher Lamb:** investigation, supervision and data curation. **Peter Irving:** writing – review and editing, supervision. **Laura Fachal:** supervision. **Gareth Walker:** conceptualization, funding acquisition, writing – review and editing. **Rachel Palmer:** validation, writing – review and editing. **Nick Kennedy:** supervision, software, conceptualization, funding acquisition, formal analysis. **Jayne Houghton:** supervision, software, project administration. **Chris Hyde:** funding acquisition, conceptualization, investigation, validation, visualization, writing – review and editing, supervision. **Miles Parkes:** supervision, methodology, writing – review and editing, writing – original draft. **James Goodhand:** investigation, conceptualization, funding acquisition, writing – original draft, methodology, writing – review and editing, visualization, formal analysis, supervision. **Tariq Ahmad:** conceptualization, investigation, funding acquisition, writing – original draft, writing – review and editing, visualization, methodology, formal analysis, supervision.

## Disclosure

Conference Presentation: Plenary presentation—2024 European Crohn's and Colitis Organisation Congress, Stockholm, Sweden. Plenary presentation—2024 British Society of Gastroenterology, Birmingham, UK. Poster presentation—2024 Digestive Diseases Week, Washington DC, USA.

## Conflicts of Interest

Karishma Sethi‐Aora has received speaker fees from Celltrion, Galapagos, Janssen, Takeda and Ferring and manuscript writing fees from Elsevier, and travel and meeting reimbursement fees from Janssen and Takeda. Shaji Sebastian has received consulting fees from Takeda, AbbVie, Merck, Ferring, Pharmacocosmos, Warner Chilcott, Janssen, Falk Pharma, Biohit, TriGenix, Celgene, and Tillots Pharma; payment or honoraria from AbbVie, Takeda, Celltrion, Pfizer, Biogen, AbbVie, Janssen, Merck, Warner Chilcott, Falk Pharma, and Janssen; is chair of the Research Committee of the British Society of Gastroenterology; is chair of the Clinical Research Committee of the European Colitis and Crohns Organisation; and is co‐director of research for the South Asian IBD Alliance. Peter Irving reports research grants from Celltrion, MSD, Takeda, Pfizer, Galapagos; lecture fees from Lecture fees: AbbVie, BMS, Celgene, Celltrion, Falk Pharma, Ferring, Galapagos, Gilead, MSD, Janssen, Lilly, Pfizer, Takeda, Tillotts, Sapphire Medical, Sandoz, Shire, Warner Chilcott; Financial support for research: Celltrion, MSD, Pfizer, Takeda, Galapagos; advisory fees from AbbVie, Arena, Boehringer‐Ingelheim, Boomerang Medical, BMS, Celgene, Celltrion, Elasmogen, Endpoint Health, Genentech, Gilead, Hospira, Janssen, Lilly, MSD, Pfizer, Pharmacosmos, Prometheus, Roche, Sandoz, Samsung Bioepis, Takeda, Topivert, VH2, Vifor Pharma, Warner Chilcott outside the submitted work. Chris Lamb acknowledges research support from the NIHR Newcastle Biomedical Research Centre, Medical Research Council, The Leona M. and Harry B. Helmsley Charitable Trust, Crohn’s & Colitis UK, EU Innovative Medicines Initiative, Wellcome Trust, Open Targets, European Bioinformatics Institute (EMBL‐EBI), Janssen, Takeda, Abbvie, AstraZeneca, Eli Lilly, Orion, Pfizer, Roche, Sanofi Aventis, UCB, Biogen, Genentech, Bristol Myers Squibb (BMS), GSK and Merck Sharp and Dohme (MSD); has undertaken consultancy for Janssen and BMS; has received honoraria for development and/or delivery of education from Takeda, Ferring, Janssen, Dr Falk, and Nordic Pharma; and has received conference attendance support from Tillotts Pharma UK, Janssen, British Society of Gastroenterology (BSG), International Organisation of IBD (IOIBD) and the European Crohn’s & Colitis Organisation (ECCO). Nick Kennedy reports institutional grants or contracts from AbbVie, Biogen, Celgene, Celltrion, Galapagos, MSD, Napp, Pfizer, Pharmacosmos, Roche, and Takeda; personal consulting fees from Amgen, Bristol Myers Squibb, Celltrion, Falk, Galapagos, Janssen, Pfizer, Pharmacosmos, Takeda, and Tillotts. Personal payments or honoraria from Amgen, Celltrion, Falk, Galapagos, Janssen, Pharmacosmos, Galapagos, Takeda, and Tillotts; support for attending meetings and/or travel from AbbVie, Falk, Janssen, and Pharmacosmos; participation in the Data Monitoring Committee for BEACON study; and Chair of British Society of Gastroenterology IBD Clinical Research Group. Miles Parkes reports grant support from Pfizer, Takeda, Astra‐Zeneca, Gilead, Galapagos, and Lilly. Tariq Ahmad reports institutional grants or contracts from Nova Pharmaceuticals, Astra‐Zeneca; consulting fees from Amgen, Celltrion, Janssen, Eli Lilly; support for attending meetings and/or travel from Tillotts and Celltrion Healthcare. All other authors make no declarations.

## Supporting information


Appendix S1.

Appendix S2.

## Data Availability

Individual participant de‐identified raw data and a data dictionary defining each field in the set will be available on application from the IBD Bioresource. The data will be available after the application has been reviewed by the NIHR data access committee. Proposals should be direct to the IBD Bioresource.
