# Supplementary material for: Clinical Utility and Cost‐Effectiveness of Pretreatment NUDT15 Pharmacogenetic Testing to Prevent Thiopurine‐Induced Myelosuppression: A Genotype‐First Reverse Phenotyping Cohort Study Within the UK NIHR Inflammatory Bowel Disease Bioresource
Source: Aliment Pharmacol Ther. 2025 Jun 23;62(6):630–45. doi: 10.1111/apt.70232 (PMC12395891; doi:10.1111/apt.70232)
Supplement: Supplementary file 1 — Appendix S1. [file APT-62-630-s001.docx]

**Supplementary appendix: '**Clinical utility and cost-effectiveness of pretreatment NUDT15 pharmacogenetic testing to prevent thiopurine-induced myelosuppression: a genotype-first reverse phenotyping cohort study within the UK NIHR inflammatory bowel disease Bioresource**’**

**Table of contents**

[Supplementary figures 3](#_Toc201251852)

[Figure S1 Plot of *NUDT15* variants and their associated functional status reported by the Clinical Pharmacogenetics Implementation Consortium (CPIC)^1^ and allele frequency in European and South Asian populations 3](#_Toc201251853)

[Figure S2 Numbers of patients with *NUDT15* variants alleles identified in the UK NIHR IBD Bioresource stratified by genetic ancestry 4](#_Toc201251854)

[Figure S3 Time to myelosuppression stratified by *NUDT15* variant status 5](#_Toc201251855)

[Figure S4 Time to myelosuppression stratified by number of *NUDT15* and *TPMT* variants present 6](#_Toc201251856)

[Figure S5 Time to myelosuppression in *NUDT15* heterozygotes stratified by most common *NUDT15* alleles 7](#_Toc201251857)

[Figure S6 Time to severe myelosuppression in *NUDT15* heterozygotes stratified by most common *NUDT15* alleles 8](#_Toc201251858)

[Figure S7 Time to myelosuppression stratified by weight-adjusted azathioprine equivalent dose 9](#_Toc201251859)

[Figure S8 Strategy 1 - Current practice: TPMT enzyme activity testing 10](#_Toc201251860)

[Figure S9 Strategy 2 - TPMT enzyme activity testing and *NUDT15* genotype testing 10](#_Toc201251861)

[Figure S10 Strategy 3 - *TPMT* and *NUDT15* genotype testing 11](#_Toc201251862)

[Figure S11 Strategy 4 – Thiopurine avoidance 11](#_Toc201251863)

[Supplementary tables 12](#_Toc201251864)

[Table S1 Proportion of patients receiving different alternative advanced therapies 12](#_Toc201251865)

[Table S2- Thiopurine treatment and monitoring costs per year for the South Asian cohort, including those on combination therapy with infliximab 12](#_Toc201251866)

[Table S3 Cost effectiveness calculations based on reducing blood test monitoring from 8 to 6 tests in the first year of treatment with a thiopurine for those with no *NUDT15* or *TPMT* variants 13](#_Toc201251867)

## Supplementary figures

Figure S1 Plot of *NUDT15* variants and their associated functional status reported by the Clinical Pharmacogenetics Implementation Consortium (CPIC)^1^ and allele frequency in European and South Asian populations

| Allele | *NUDT15* haplotype   \| Exon 1 \| Exon 2 \| Exon 3 \| \| --- \| --- \| --- \| | Function based on current CPIC definition | Frequency in European populations | Frequency in South Asian populations |
| --- | --- | --- | --- | --- | --- | --- | --- |
| *1 | 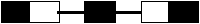 | Normal function | 99.3% | 93.0% |
| *2 | 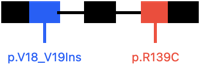 | No function | <0.001% | <0.001% |
| *3 | 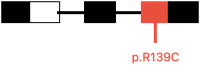 | No function | 0.20% | 6.7% |
| *4 | 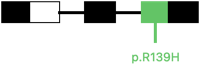 | Uncertain function | 0.003% | 0.003% |
| *5 | 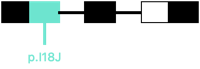 | Uncertain function | 0.001% | 0.045% |
| *6 | 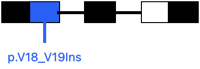 | Uncertain function | 0.3% | 0.2% |
| *9 | 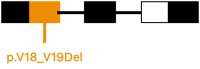 | No function | 0.18% | 0.05% |

### Figure S2 Numbers of patients with *NUDT15* variants alleles identified in the UK NIHR IBD Bioresource stratified by genetic ancestry


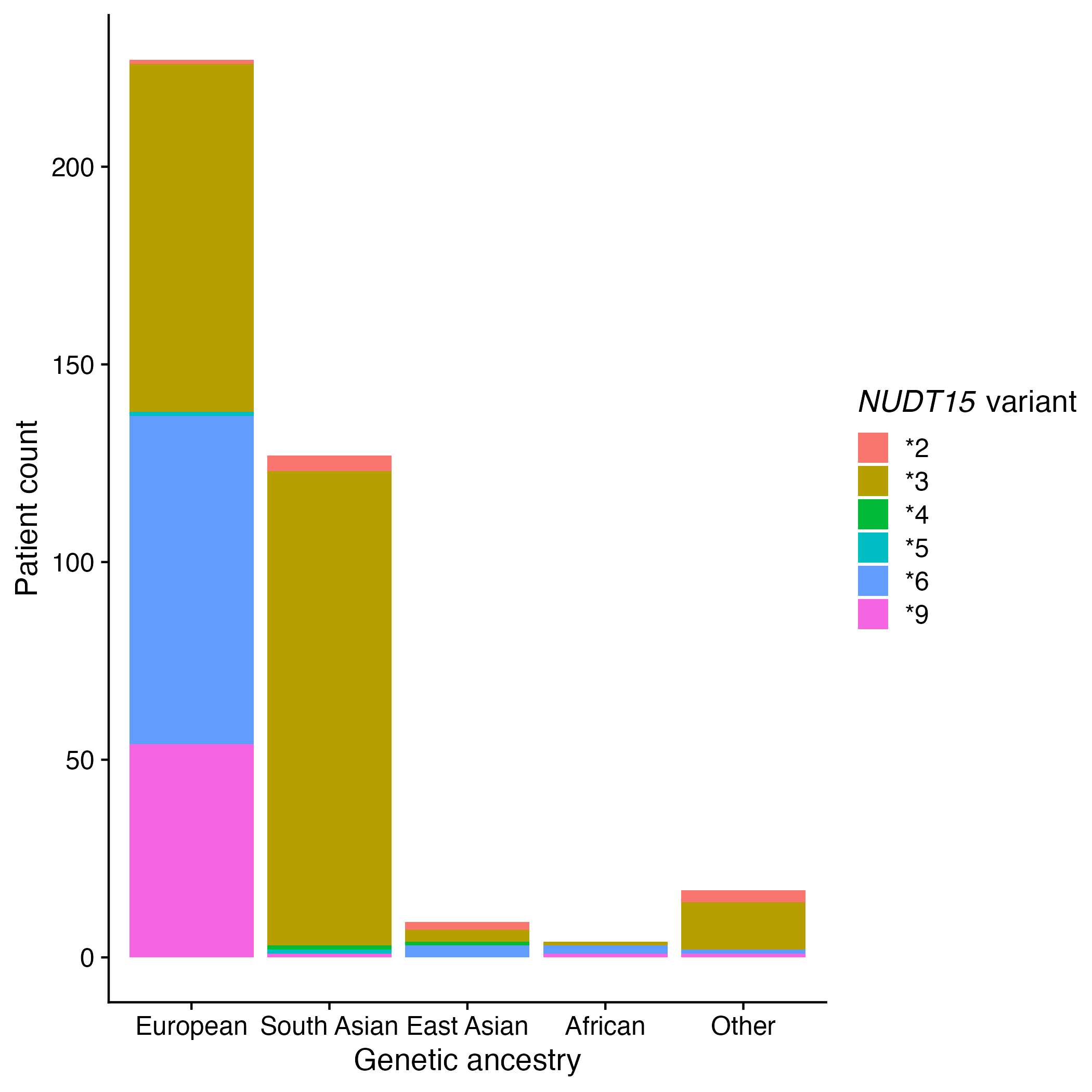


### Figure S3 Time to myelosuppression stratified by *NUDT15* variant status


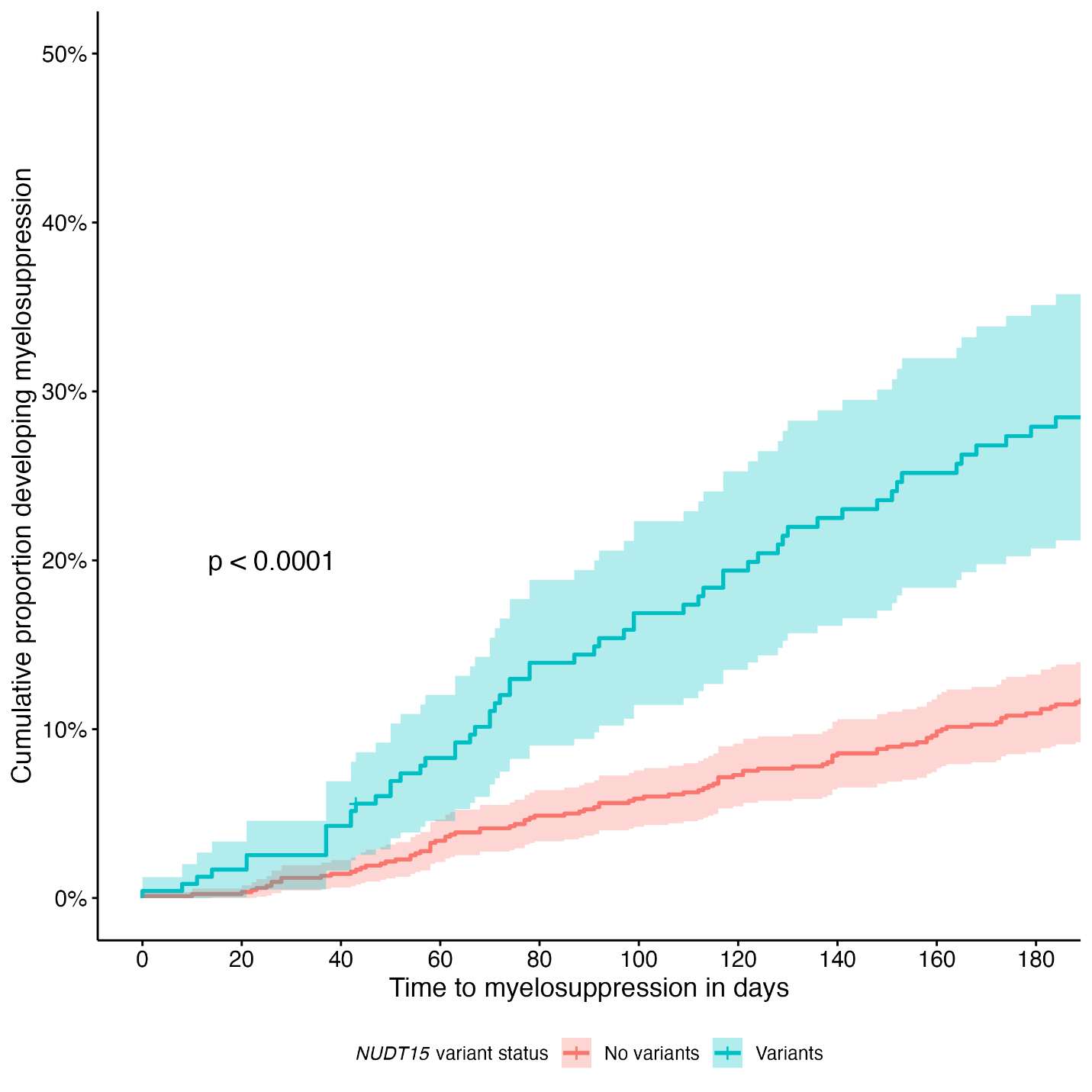
Myelosuppression was defined as an episode where WCC <3.5x10^9^/L or neutrophil count <2.0x10^9^/L or in the absence of blood test data a decision to either dose reduce or stop the thiopurine due to myelosuppression.

### Figure S4 Time to myelosuppression stratified by number of *NUDT15* and *TPMT* variants present


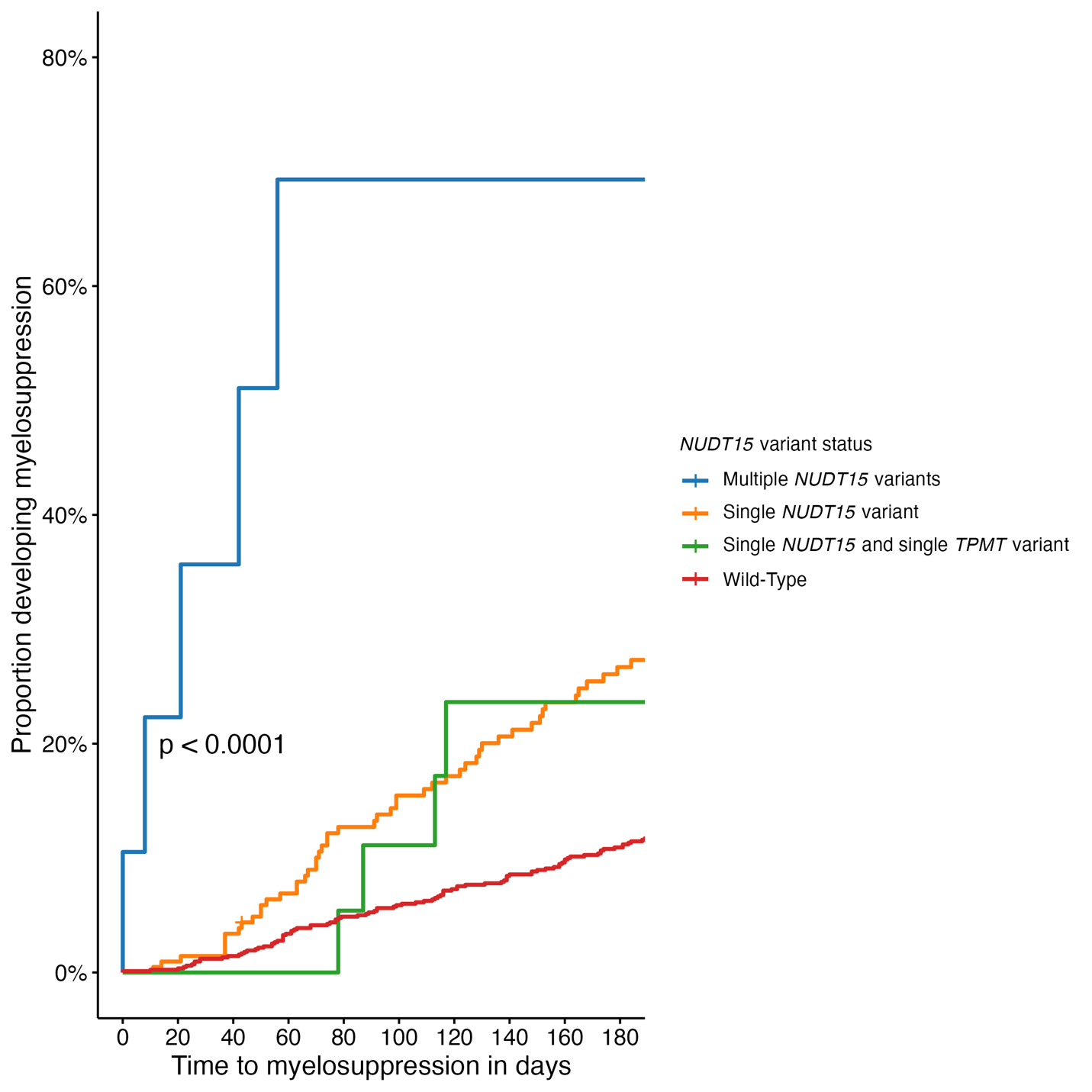


Myelosuppression was defined as an episode where WCC <3.5x10^9^/L or neutrophil count <2.0x10^9^/L or in the absence of blood test data a decision to either dose reduce or stop the thiopurine due to myelosuppression.

### Figure S5 Time to myelosuppression in *NUDT15* heterozygotes stratified by most common *NUDT15* alleles


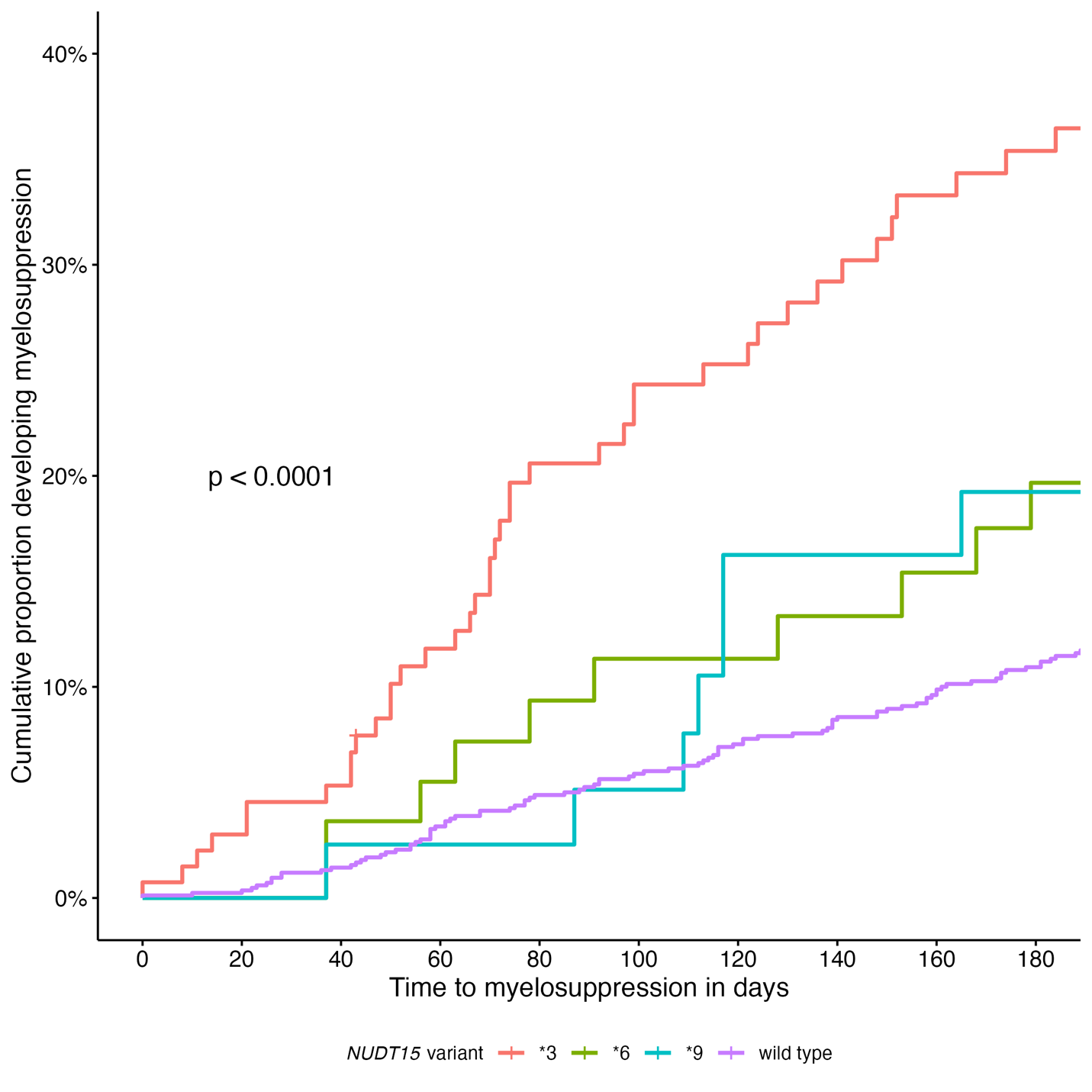


Myelosuppression was defined as an episode where WCC <3.5x10^9^/L or neutrophil count <2.0x10^9^/L or in the absence of blood test data a decision to either dose reduce or stop the thiopurine due to myelosuppression.

### Figure S6 Time to severe myelosuppression in *NUDT15* heterozygotes stratified by most common *NUDT15* alleles


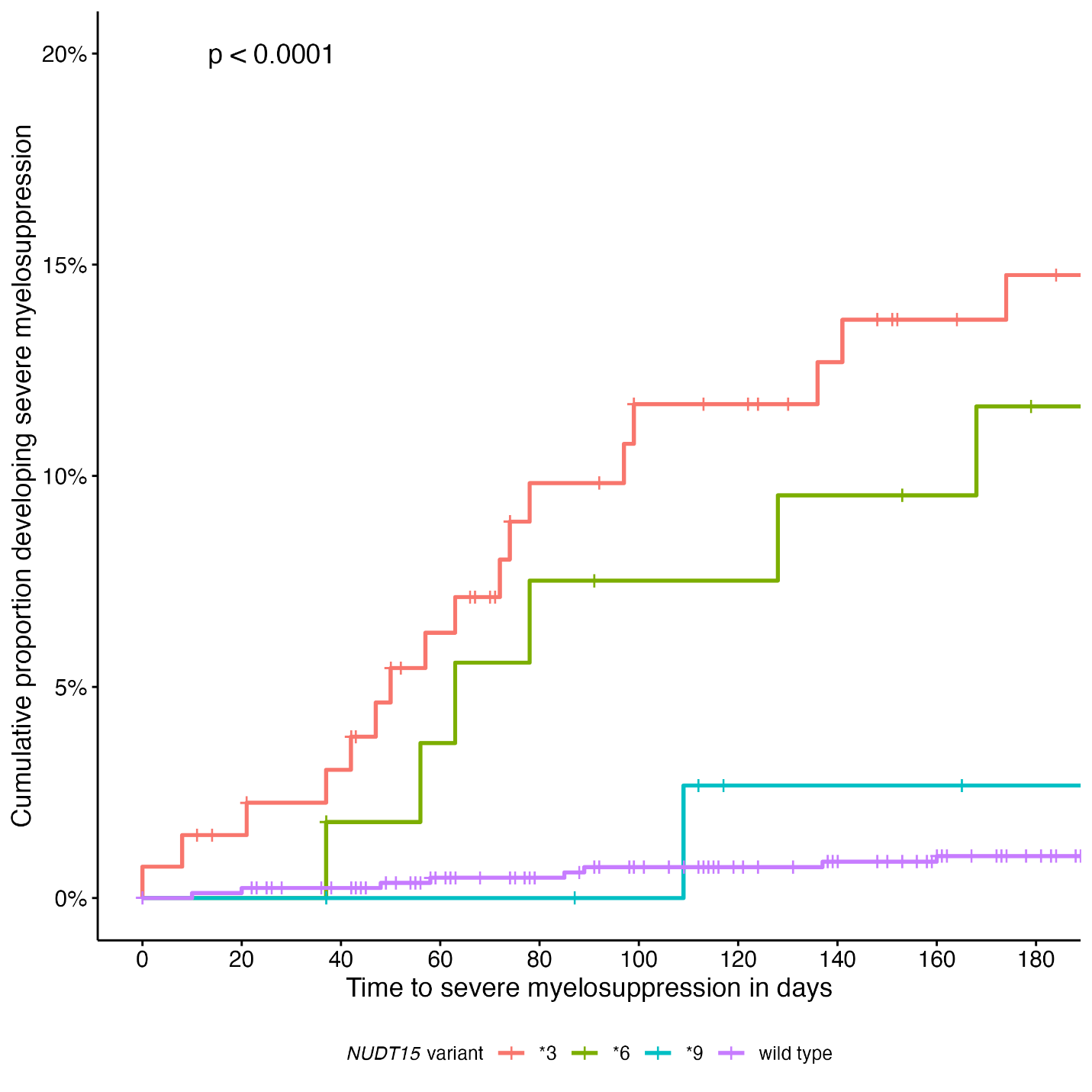


Severe myelosuppression defined as an episode where WCC <2.5x10^9^/L or neutrophil count <1.0x10^9^/L and a decision to either dose reduce or stop the thiopurine.

### Figure S7 Time to myelosuppression stratified by weight-adjusted azathioprine equivalent dose


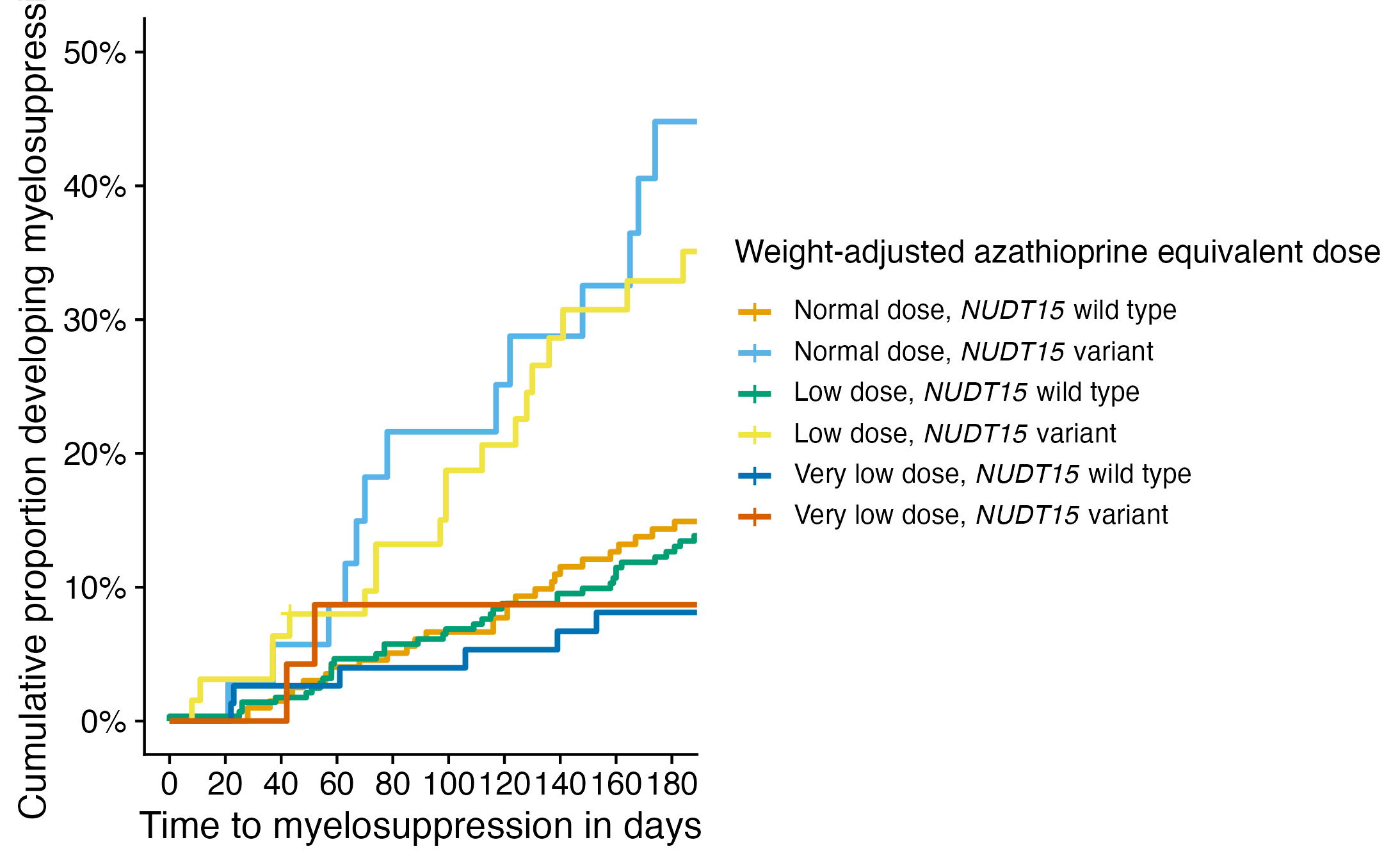


Myelosuppression was defined as an episode where WCC <3.5x10^9^/L or neutrophil count <2.0x10^9^/L or in the absence of blood test data a decision to either dose reduce or stop the thiopurine due to myelosuppression. Normal dose was a weight-adjusted azathioprine equivalent dose of greater than 2 mg/kg/day; low dose was a weight-adjusted azathioprine equivalent dose of between 1-2 mg/kg/day and very low dose weight-adjusted azathioprine equivalent dose of less than 1 mg/kg/day.

### Figure S8 Strategy 1 - Current practice: TPMT enzyme activity testing


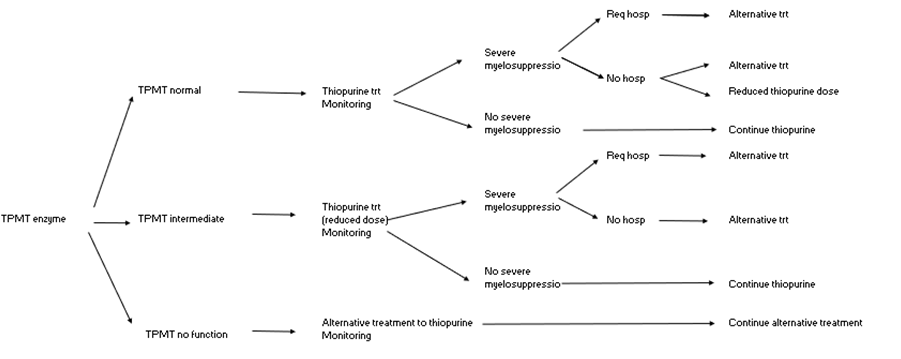


### Figure S9 Strategy 2 - TPMT enzyme activity testing and *NUDT15* genotype testing


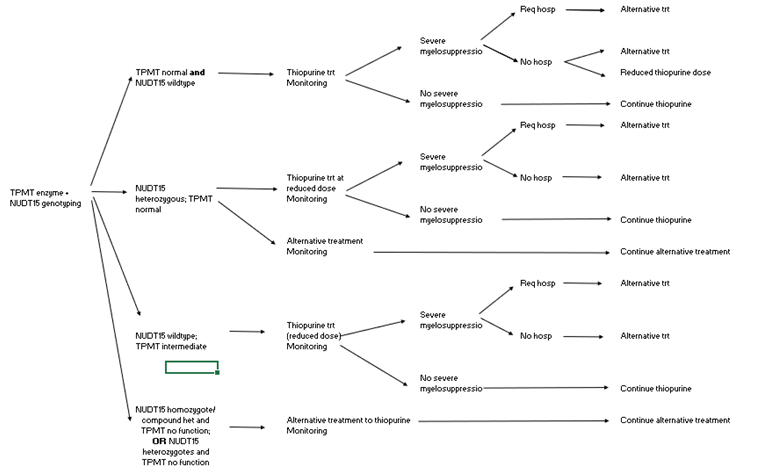


### Figure S10 Strategy 3 - *TPMT* and *NUDT15* genotype testing


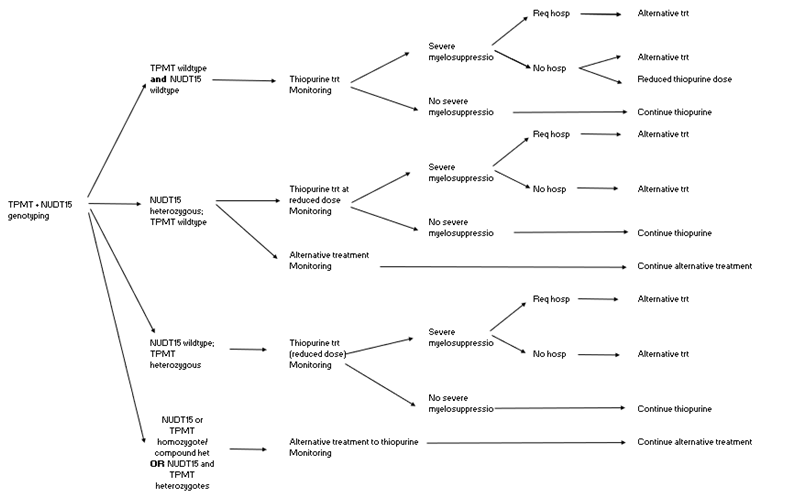


### Figure S11 Strategy 4 – Thiopurine avoidance


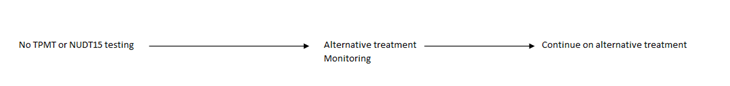


## Supplementary tables

### Table S1 Proportion of patients receiving different alternative advanced therapies

| **Treatment** | **Proportion receiving treatment** |
| --- | --- |
| **Ulcerative colitis** | |
| filgotinib | 0.18 |
| upadacitinib | 0.42 |
| vedolizumab | 0.2 |
| adalimumab | 0.1 |
| infliximab | 0.1 |
| **Crohn’s disease** | |
| adalimumab | 0.623 |
| infliximab | 0.267 |
| upadacitinib | 0.055 |
| ustekinumab | 0.055 |

### Table S2- Thiopurine treatment and monitoring costs per year for the South Asian cohort, including those on combination therapy with infliximab

| **Patient group** | **Treatment*** | | | **Monitoring** |
| --- | --- | --- | --- | --- |
|  | **General population** | **European ancestry subgroup** | **South Asian ancestry subgroup** |  |
| Recommended dose - no severe TIM** | £1,750 | £1,750 | £1,741 | £165.09 +  £131.82 |
| Reduced dose – no severe TIM | £1,733 | £1,733 | £1,732 | £165.09 +  £131.82 |
| Recommended dose - severe TIM | £1,154 | £1,154 | £1,151 | £67.35 + £131.82 |

* Assuming mean weight as given above and azathioprine cost of £4.32 for pack of 100 25mg tablets and £2.89 for pack of 100 50mg tablets (BNF), and mercaptopurine cost of £8.42 for pack of 25 50mg tablets. ** Note that for individuals who are TPMT and NUDT15 wildtype, it is assumed that they receive just 6 monitoring tests within the first year.

### Table S3 Cost effectiveness calculations based on reducing blood test monitoring from 8 to 6 tests in the first year of treatment with a thiopurine for those with no *NUDT15* or *TPMT* variants

| **Strategy** | **% severe TIM** | **QALYs** | **Total costs** | **Compared to current practice** | | | | |
| --- | --- | --- | --- | --- | --- | --- | --- | --- |
|  |  |  |  | **Incremental severe TIM events** | **Incremental QALYs** | **Incremental costs** | **ICER (per severe TIM avoided)** | **ICER (per QALY gained)** |
| **General population** | | | | | | | | |
| 1) TPMT enzyme testing (Current practice) | 1.20% | 0.69957 | £2,007 |  | | | | |
| 2) TPMT enzyme and *NUDT15* genotyping | 1.17% | 0.69959 | £2,009 | -0.04% | 0.00001 | £2 | £6,447 | £182,186 |
| 3) *NUDT15* and *TPMT* genotyping | 1.17% | 0.69959 | £2,004 | -0.04% | 0.00001 | -£3 | Dominates current practice | |
| 4) Alternative treatment | 0% | 0.70000 | £4,743 | -1.20% | 0.00043 | £2,737 | £227,610 | £6.43 million |
| **European ancestry sub-population** | | | | | | | | |
| 1) TPMT enzyme testing (Current practice) | 1.17% | 0.69959 | £2,006 |  | | | | |
| 2) TPMT enzyme and *NUDT15* genotyping | 1.14% | 0.69960 | £2,005 | -0.04% | 0.00001 | -£1 | Dominates current practice | |
| 3) *NUDT15* and *TPMT* genotyping | 1.14% | 0.69960 | £2,000 | -0.04% | 0.00001 | -£6 |  |  |
| 4) Alternative treatment | 0% | 0.70000 | £4,811 | -1.17% | 0.00041 | £2,805 | £239,482 | £6.77 million |
| **South Asian ancestry sub-population** | | | | | | | | |
| 1) TPMT enzyme testing (Current practice) | 2.01% | 0.69929 | £2,012 |  | | | | |
| 2) TPMT enzyme and *NUDT15* genotyping | 1.95% | 0.69931 | £2,054 | -0.06% | 0.00002 | £42 | £73,930 | £2.09 million |
| 3) *NUDT15* and *TPMT* genotyping | 1.95% | 0.69931 | £2,049 | -0.06% | 0.00002 | £36 | £64,890 | £1.83 million |
| 4) Alternative treatment | 0% | 0.70000 | £4,646 | -2.01% | 0.00071 | £2,634 | £131,326 | £3.71 million |

Reference:

1 Relling M V., Schwab M, Whirl-Carrillo M, *et al.* Clinical Pharmacogenetics Implementation Consortium Guideline for Thiopurine Dosing Based on TPMT and NUDT15 Genotypes: 2018 Update. *Clin Pharmacol Ther* 2019; 105. DOI:10.1002/cpt.1304.
