# Supplementary material for: Clinical Utility and Cost‐Effectiveness of Pretreatment NUDT15 Pharmacogenetic Testing to Prevent Thiopurine‐Induced Myelosuppression: A Genotype‐First Reverse Phenotyping Cohort Study Within the UK NIHR Inflammatory Bowel Disease Bioresource
Source: Aliment Pharmacol Ther. 2025 Jun 23;62(6):630–45. doi: 10.1111/apt.70232 (PMC12395891; doi:10.1111/apt.70232)
Supplement: Supplementary file 2 — Appendix S2. [file APT-62-630-s002.docx]

# Supplementary methods: 'Clinical utility and cost-effectiveness of pretreatment *NUDT15* pharmacogenetic testing to prevent thiopurine-induced myelosuppression: a genotype-first reverse phenotyping cohort study within the UK NIHR inflammatory bowel disease Bioresource’

## Genetic methods

### Whole genome and whole exome sequencing^1^

Whole genome sequencing was performed on the Illumina HiSeq X platform (Illulmina, San Diego, California, USA) using a combination of PCR and PCR-free library preparation protocols. Sequencing was conducted to a median depth of 18.6X. Whole exome sequencing was performed on the Illumina NovaSeq 6000 platform using the Agilent SureSelect Human Exon V5 capture set (Agilent Technologies, Santa Clara, California, USA).

DNA reads were mapped to the human genome 38 reference using Burrow-Wheller Aligner versions 0.7.12 (WGS) and 0.7.17 (WES)^2^. Variant calls were performed using the genome analysis toolkit, Best Practices-like pipeline (versions 4.0.10.1 [WGS] and 4.1.8 [WES]). Per-sample intermediate variant calling was followed by joint genotyping across the individual genome and exome cohorts.

We selected a set of ∼14,000 well-genotyped common variants to identify the genetic ancestry of individual participants using the 1000 Genomes Project derived principal components^3,4^. For genomes, we excluded samples outside of four median absolute deviations from the median point of the European ancestry cluster. For exomes, we implemented a random forest technique that classified samples based on principal components into the following genetic ancestry groups: European, African, South Asian and East Asian and other with self-reported ancestry as training labels.

A combination of hard-cutoff filters and per-ancestry/per-batch outlier filters were used to identify low-quality samples that were then excluded. We applied hard filters for sample depth (> 12x genomes, > 15x exomes), call rate (> 0.95), chimerism < 0.5 (WGS) and FREEMIX < 0.02 (WGS). We excluded genotype calls with an allelic imbalance (for heterozygotes, (allelic balance [ab] < 0.20) | (ab > 0.80)), low depth (< 2x), and low genetic quality (GQ < 20). We then performed per-ancestry and per-sequencing protocol filtering of samples falling outside 4 median absolute deviations from the median per-batch heterozygosity rate, transition/transversion ratio, number of called single nucleotide polymorphisms (SNPs) and insertion and deletion counts/ratio.

An ancestry-aware relatedness calculation (pc-relate method in Hail^5^) was used to identify related samples. As our association approach (logistic mixed-models) can control for residual relatedness, we only excluded duplicates or monozygotic twins from within the cohorts and excluded second and third degree relatives when the kinship was across the cohorts (e.g., parent in WGS, child in WES; kinship metric > 0.1 calculated via PC-Relate^5^ method using 10 principal components).

### Loop-mediated isothermal amplification (LAMP) *NUDT15* test

LAMP testing was carried out using the Human NUDT15 deficiency KIT (LC-NUDT15-LP). Whole blood was thawed and using a robot 400ml of lysis buffer was added to 2ml of blood into an Eppendorf 1ml deep well plate (Eppendorf Cat No.: 0030503104). Samples were incubated for at least 1 minute at room temperature. Assay mix (20ml) was added for each reaction (3* and 6*/9*) into a 96 well Taqman optically clear plate (Azenta Life Sciences Cat No.: 4TI-0730/C) and lysed blood sample (5ml) added. The genotyping was carried out using the QuantStudio 1 Real-time PCR platform (Thermo Fisher Scientific, Waltham, Massachusetts, USA) using the LAMP99 fast melt-QS1 profile (LaCAR MDx Technologies, Liege, Belgium; Customer area: https://www.lacar-mdx.com/customer-area/lacar/documents/m/profiles/quant-studio/qs1). Data was analysed with QuantStudio Design and Analysis Software (v1.53) (Thermo Fisher Scientific, Waltham, Massachusetts, USA) and genotypes were called using the Genefox software using predefined criteria assigned by the supplier (LaCAR MDx Technologies, Liege, Belgium https://lacar.genefox.be/#/). Samples that failed quality control checks were repeated manually.

### Sanger sequencing methods

Sanger sequence analyses were undertaken of the coding exons and flanking intronic regions of the *NUDT15* gene (NM_018283.4) and the *TPMT* gene (NM_000367.5) using standard conditions and following manufacturers’ protocols (primer sequences available on request). Sequencing reactions were run on an ABI 3730XL DNA Analyzer (Applied Biosystems, Warrington, UK) and analysed using Mutation Surveyor, v5.1.2 (SoftGenetics, State College, PA, USA).

## References

1 Sazonovs A, Stevens CR, Venkataraman GR, *et al.* Large-scale sequencing identifies multiple genes and rare variants associated with Crohn’s disease susceptibility. *Nat Genet* 2022; 54. DOI:10.1038/s41588-022-01156-2.

2 Li H, Durbin R. Fast and accurate short read alignment with Burrows-Wheeler transform. *Bioinformatics* 2009; 25. DOI:10.1093/bioinformatics/btp324.

3 Auton A, Abecasis GR, Altshuler DM, *et al.* A global reference for human genetic variation. Nature. 2015; 526. DOI:10.1038/nature15393.

4 Price AL, Patterson NJ, Plenge RM, Weinblatt ME, Shadick NA, Reich D. Principal components analysis corrects for stratification in genome-wide association studies. *Nat Genet* 2006; 38. DOI:10.1038/ng1847.

5 Broad Institute. Hail 0.2. 2025; published online April 29.
